# Donor–recipient HLA molecular mismatch and T follicular helper-related genetic variants are associated with dnDSA development after kidney transplantation

**DOI:** 10.3389/fimmu.2026.1875029

**Published:** 2026-07-06

**Authors:** Hidetoshi Shidahara, Kentaro Ide, Aiko Yamaoka, Yuki Imaoka, Seiichi Shimizu, Hiroyuki Tahara, Masahiro Ohira, Yuka Tanaka, Hideki Ohdan

**Affiliations:** 1 Department of Gastroenterological and Transplant Surgery, Graduate School of Biomedical and Health Sciences, Hiroshima University, Hiroshima, Japan; 2 Section of Clinical Laboratory, Department of Clinical Support, Hiroshima University Hospital, Hiroshima, Japan

**Keywords:** alloimmunity, *de novo* donor-specific antibody, germinal center, HLA molecular mismatch, immunogenetics, kidney transplantation, T follicular helper cell

## Abstract

**Introduction:**

*De novo* donor-specific HLA antibodies (dnDSAs) after kidney transplantation arise through coordinated antigen recognition, T-cell–dependent B-cell activation, and subsequent germinal center reactions. Donor–recipient HLA molecular mismatch reflects donor-derived antigenic disparity and is associated with dnDSA development; however, it does not fully explain interindividual variability in humoral alloimmune responses. We hypothesized that integrating HLA molecular mismatch with recipient T follicular helper (Tfh) cell-related immunogenetic variation would improve risk stratification and provide additional insight into variability in alloantibody responses.

**Methods:**

In this retrospective single-center cohort study, we analyzed 210 kidney transplant recipients who underwent transplantation between 2011 and 2024. Donor–recipient HLA molecular mismatch loads were quantified using eplet mismatch analysis, Predicted Indirectly ReCognizable HLA Epitopes–T cell (PIRCHE-T2), and HLA Epitope Mismatch Algorithm (HLA-EMMA). Recipient single-nucleotide polymorphisms in Tfh-related genes (*CXCR5* rs3922 and *CTLA4* rs231775) were genotyped. The primary endpoint was class II dnDSA development, defined as newly detected donor-specific antibodies with a normalized mean fluorescence intensity >1, 000 and persistence confirmed at least six months later. Associations between candidate variables and class II dnDSA development were assessed using Firth penalized logistic regression.

**Results:**

During follow-up, 20 recipients (9.5%) developed class II dnDSAs. Higher class II molecular mismatch loads were significantly associated with dnDSA development, with HLA-EMMA showing the strongest association among the evaluated metrics. In multivariable analysis, high HLA-EMMA class II load and Tfh cell-related immunogenetic variation were independently associated with class II dnDSA development. Recipients with both high HLA-EMMA class II load and Tfh-related polymorphisms showed the highest cumulative incidence of dnDSA development.

**Conclusion:**

These findings suggest that donor-derived structural antigenicity and recipient Tfh-related immunogenetic variation are associated with the development of class II dnDSAs after kidney transplantation. The identified thresholds and integrated risk stratification approach are exploratory and require validation in independent cohorts before clinical application of the model.

## 1 Introduction

The development of *de novo* donor-specific antibodies (dnDSAs) after kidney transplantation (KT) is a hallmark of humoral alloimmunity and represents a critical step toward antibody-mediated rejection (AMR) (1–4). From an immunological perspective, alloantibody generation is initiated by recognition of donor-derived alloantigens, followed by antigen processing and presentation, activation of CD4^+^ T cells, and subsequent T-cell–dependent B-cell responses culminating in germinal center reactions (5–7). These coordinated processes ultimately result in the production of class-switched, high-affinity antibodies capable of mediating allograft injury (6, 8).

Recent advances in human leukocyte antigen (HLA) immunogenetics have enabled more precise quantification of donor–recipient immunological disparities beyond conventional antigen-level matching. Molecular mismatch algorithms, including eplet mismatch analysis (EpMM), Predicted Indirectly ReCognizable HLA Epitopes–T-cell (PIRCHE-T2), and the HLA Epitope Mismatch Algorithm (HLA-EMMA), capture complementary aspects of alloantigen recognition (9–18). EpMM reflects structural differences in B-cell–accessible epitopes (9, 13–15), whereas PIRCHE-T2 estimates the repertoire of donor-derived peptides that may be indirectly presented to CD4^+^ T cells (10–12, 17, 18). HLA-EMMA further evaluates solvent-accessible amino acid mismatches at the molecular surface, potentially providing a higher-resolution representation of structural antigenicity relevant to antibody recognition (16). Collectively, these approaches have improved the understanding of donor immunogenicity and its association with dnDSA development.

However, despite these methodological advances, molecular mismatch alone does not fully explain the interindividual variability in humoral alloimmune responses after transplantation. Recipients with comparable HLA mismatch levels may exhibit divergent outcomes, with some developing robust alloantibody responses while others remain immunologically quiescent (19, 20). This discrepancy suggests that donor-derived antigenicity represents only one component of humoral alloimmune responses and highlights the importance of recipient-dependent immune regulatory mechanisms.

T follicular helper (Tfh) cells play a central role in orchestrating germinal center reactions and humoral immune responses. These cells express C-X-C motif chemokine receptor 5 (CXCR5), which directs their migration into B-cell follicles, where germinal center reactions occur (21). They also produce interleukin-21, a key cytokine that promotes B-cell activation, differentiation, class-switch recombination, and affinity maturation (22). In addition, Tfh cells have been implicated in the regulation of alloantibody responses after transplantation (23). The magnitude and quality of Tfh-mediated help are critical determinants of antibody production, and dysregulation of Tfh responses has been implicated in autoimmune and alloimmune conditions (7). Accordingly, interindividual differences in Tfh cell differentiation, trafficking, and function may contribute to the variability in alloantibody responses after transplantation.

Genetic polymorphisms affecting immune regulatory pathways are a potential source of this variability. *CXCR5* is essential for Tfh cell migration into B-cell follicles, whereas cytotoxic T-lymphocyte–associated antigen 4 (*CTLA4*) functions as a key negative regulator of T-cell activation (24). Variations in these genes have been proposed to influence T-cell activation thresholds, Tfh cell differentiation and function, and ultimately the efficiency of T-cell–dependent B-cell responses. We have previously reported associations between Tfh-related genetic polymorphisms and dnDSA development (25, 26), supporting the concept that host immunogenetic background modulates humoral alloimmunity.

Based on these considerations, we hypothesized that dnDSA development would be associated with the interaction between donor-derived HLA molecular mismatch and recipient immunogenetic susceptibility related to Tfh cell biology. Although these factors have been individually associated with dnDSA development, their combined contribution to humoral alloimmunity remains incompletely understood. In this study, we investigated the combined association with molecular mismatch metrics and Tfh-related genetic polymorphisms on dnDSA development in kidney transplant recipients. By integrating structural measures of donor immunogenicity with recipient immune responsiveness, we aimed to provide a more comprehensive immunogenetic framework for understanding the heterogeneity of alloantibody responses after transplantation.

## 2 Materials and methods

### 2.1 Patients

This single-center retrospective cohort study included patients who underwent KT between May 2011 and June 2024 at Hiroshima University Hospital. Patients were excluded if they had identical donor–recipient HLA profiles, lacked genomic DNA samples, or had a follow-up period of <18 months. Recipients with ABO-incompatible transplantation and those with preformed DSA were included to reflect real-world clinical kidney transplantation practices. Clinical data, including age, sex, primary disease, donor relationship, dialysis duration, and donor–recipient HLA mismatch, were collected at the time of transplantation. This study was conducted in accordance with the Declaration of Helsinki and approved by the Institutional Review Board of Hiroshima University (approval numbers: C2007–4017 and E2018-9223). Written informed consent was obtained from all participants. This study is reported in accordance with the Strengthening the Reporting of Observational Studies in Epidemiology (STROBE) guidelines.

### 2.2 HLA typing, crossmatching, and anti-HLA testing

HLA typing was performed at the HLA-A, -B, -C, -DRB1, and -DQB1 loci using xMAP technology (Luminex Corporation, Austin, TX, USA) in combination with polymerase chain reaction sequence-specific oligonucleotide probes (Wakunaga, Hiroshima, Japan). High-resolution HLA typing data were unavailable for any of the evaluated loci in the present cohort. This system detects alleles with a frequency ≥0.1% based on population allele frequency data derived from the Japanese population. Accordingly, the allele level information required for molecular mismatch analyses was estimated using established Japanese population-based haplotype frequency datasets. Specifically, DRB3/4/5 and DQA1 alleles were inferred using a local haplotype frequency dataset derived from 916 unrelated Japanese individuals (27), and ambiguous HLA assignments were resolved by selecting the most probable allele combinations according to Japanese population-based haplotype frequencies. The inferred allele assignments were subsequently used for molecular mismatch calculations, including eplet mismatch analysis, PIRCHE-T2, and HLA-EMMA. Complement-dependent cytotoxicity crossmatch and flow cytometric crossmatch were performed as previously described (28). Immunocomplex capture fluorescence analysis (WAKFlow HLA antibody class I&II, Wakunaga Pharmaceutical Co., Ltd., Japan) was additionally used as an alternative crossmatch assay in accordance with the manufacturer’s instructions.

Anti-HLA antibody screening was routinely performed before transplantation, at 3 months after transplantation, and annually thereafter in all recipients. Additional testing was performed when clinically indicated, including episodes of graft dysfunction. Recipients with preformed anti-HLA antibodies underwent additional surveillance at 1 month and 6 months after transplantation because of their higher immunologic risk. Thus, although all recipients underwent standardized annual surveillance, the overall frequency of antibody monitoring was not completely uniform across the cohort because additional assessments were performed according to clinical indications and immunologic risk status. Single-antigen bead assays (LABScreen, One Lambda, Canoga Park, CA, USA) were performed using a Luminex platform. dnDSAs were defined as DSAs that were absent before transplantation and newly detected thereafter, with a normalized mean fluorescence intensity (nMFI) >1, 000. Persistence was defined as repeat positivity at least six months after initial detection. In recipients with preformed DSA, post-transplant antibodies directed against donor HLA specificities absent before transplantation were classified as dnDSA, whereas recurrence of previously identified DSA specificities was not considered *de novo* antibody development. In patients with multiple dnDSAs, the immunodominant DSA (iDSA), defined as the antibody with the highest nMFI, was used as the representative antibody for analysis.

### 2.3 HLA molecular mismatch analysis

Antibody-verified eplet mismatches (EpMMs) for HLA class I (HLA-A, -B, and -C) and class II (HLA-DRB1, -DRB3/4/5, and -DQ) were calculated using HLA Fusion software version 4.7.1 (One Lambda). Locus-specific EpMMs were denoted as EpMM-A, -B, -C, -DRB1, -DRB3/4/5, and -DQ. The sum of EpMM-DRB1, EpMM-DRB3/4/5, and EpMM-DQ was defined as the EpMM class II load.

The Predicted Indirectly ReCognizable HLA Epitopes–T cell (PIRCHE-T2) score was calculated using the PIRCHE algorithm (IMGT/HLA version 4.2). Scores were computed for each locus and summarized as PIRCHE-A, -B, -C, -DR (DRB1+DRB3/4/5), and -DQ (DQA1+DQB1). The sum of PIRCHE-DR and PIRCHE-DQ was defined as the PIRCHE class II score.

HLA Epitope Mismatch Algorithm (HLA-EMMA) version 1.06 (IMGT/HLA database version 3.47) was used to assess amino acid-level mismatches for HLA class I (HLA-A, -B, and -C) and class II (HLA-DR, -DQA1, and -DQB1). Solvent-accessible amino acid mismatches were quantified and denoted as EMMA-A, -B, -C, -DR, and -DQ (DQA1+DQB1). The sum of EMMA-DR and EMMA-DQ was defined as the EMMA class II load.

### 2.4 DNA extraction and genotyping

Two Tfh cell-related single-nucleotide polymorphisms (SNPs), rs3922 in *CXCR5* and rs231775 in *CTLA4*, were selected *a priori* based on our previous studies demonstrating associations with dnDSA development after KT (25, 29) and their biological relevance to Tfh-cell regulation. Because only two prespecified candidate SNPs were evaluated, correction for multiple testing was not applied. Genomic DNA was extracted from peripheral blood mononuclear cells using the QIAamp DNA Blood Mini Kit (QIAGEN, Hilden, Germany). SNP genotyping was performed using TaqMan SNP genotyping assays (Thermo Fisher Scientific, Waltham, MA, USA) according to the manufacturer’s protocol. Briefly, allele-specific TaqMan probes containing distinct fluorescent dyes, together with a PCR primer pair, were used to detect the target SNPs. Recipients carrying the rs3922 G allele (GG or AG genotype) together with the rs231775 GG genotype were classified as having a Tfh-prone genetic profile (Tfh high) based on our previous findings (25, 29). Genotype distributions were assessed for conformity to Hardy–Weinberg equilibrium using exact tests.

### 2.5 Desensitization protocol and immunosuppressive regimen

Patients with ABO-incompatible transplants received desensitization therapy consisting of a single dose of rituximab (375 mg/m²) administered two weeks before transplantation, followed by initiation of calcineurin inhibitors (cyclosporine or tacrolimus) and mycophenolate mofetil. Double-filtration plasmapheresis (0–5 sessions) was performed to achieve ≥16-fold reduction in isoagglutinin titers before transplantation (30). Patients with preformed DSAs (≥3, 000 nMFI) and positive crossmatches underwent desensitization with rituximab and plasmapheresis. Transplantation was performed after achieving crossmatch negativity and DSA reduction (<1, 000 nMFI). In refractory cases, bortezomib-based therapy was administered according to a standardized protocol (31). All patients received standard immunosuppressive therapy consisting of induction with basiliximab or anti-thymocyte globulin induction, followed by maintenance therapy with calcineurin inhibitors, mycophenolate mofetil, and corticosteroids. The target trough levels were adjusted according to institutional protocols (31).

### 2.6 Statistical analysis

Statistical analyses were performed using JMP version 18 (SAS Institute, Cary, NC, USA). Continuous variables are presented as medians (interquartile range or ranges, as appropriate) and compared using Student’s t-test or Mann–Whitney U test, as appropriate, based on data distribution. Categorical variables were compared using the chi-square test or Fisher’s exact test. Univariable and multivariable analyses were performed using Firth penalized logistic regression with the logistf package in R version 4.5.2 (R Foundation for Statistical Computing, Vienna, Austria) to reduce small-sample bias and improve estimation stability under conditions of limited event frequency. Because only 20 recipients developed class II dnDSA, multivariable analyses were restricted to minimize overfitting and unstable parameter estimates. Collinearity among molecular mismatch metrics (EpMM, PIRCHE-T2, and HLA-EMMA) was evaluated using Spearman correlation analysis. Given the substantial overlap among these variables, HLA-EMMA was selected as the representative molecular mismatch metric for the primary multivariable model. The final multivariable model included two variables (HLA-EMMA class II and Tfh-high status), resulting in an events-per-variable (EPV) ratio of 10 (20 events for 2 variables). Continuous-variable analyses were additionally performed using univariable Firth penalized logistic regression per standard deviation (SD) increase for each molecular mismatch metric. Sensitivity analyses were also performed after excluding ABO-incompatible recipients and recipients with preformed DSA. Time-to-event outcomes were analyzed using the Kaplan–Meier method and compared using the log-rank test. Discriminatory performance was assessed using receiver operating characteristic analysis, and optimal cutoff values were determined using the Youden index. A two-sided P value of <0.05 was considered statistically significant.

## 3 Results

### 3.1 Patient characteristics and incidence of dnDSA

Between May 2011 and June 2024, 234 KTs were performed at Hiroshima University Hospital. Of these, 24 patients were excluded due to identical donor–recipient HLA profiles (n = 8), lack of genomic DNA (n = 4), or a follow-up period of <18 months (n = 12). A total of 210 patients were included in the final analysis. During follow-up, 25 patients (11.9%) developed dnDSAs. The median time to dnDSA development was 3.9 years (range 0.3–9.9). Among these patients, immunodominant DSAs (iDSAs) were directed against HLA class I in 5 and HLA class II in 20 cases. The median time to dnDSA development was 4.0 years (3.3–8.8) for class I and 2.7 years (0.3–9.9) for class II, with no significant difference between the groups (P = 0.209). The median nMFI at first detection was significantly higher for class II iDSAs than that for class I iDSAs [6, 754 (1, 238–30, 262) vs. 2, 147 (1, 230–2, 688); P = 0.016]. Given the predominance and established clinical relevance of HLA class II dnDSAs (32), together with the small number of class I dnDSAs (n = 5), subsequent analyses focused on class II dnDSAs.

### 3.2 Baseline and immunological characteristics according to class II dnDSA status

Baseline characteristics of the patients stratified by the presence of class II dnDSAs are summarized in **Table 1**. No significant differences were observed between groups with respect to age, sex, primary disease, dialysis duration, history of sensitization, donor–recipient relationship, ABO compatibility, presence of preformed DSA, choice of calcineurin inhibitor, or cold ischemia time. A history of T-cell–mediated rejection was numerically more frequent in the class II dnDSA-positive group (10.0% vs. 1.6%). However, this association did not reach statistical significance in univariable logistic regression analysis [odds ratio (OR), 6.93; 95% confidence interval (CI), 0.87–44.48; P = 0.065]. We also evaluated clinically relevant alloimmune outcomes according to class II dnDSA status. Among recipients with class II dnDSA positivity, antibody-mediated rejection (AMR) and biopsy-proven rejection occurred in 3 (15.0%) and 2 (10.0%) patients, respectively, whereas among recipients without class II dnDSA, these events occurred in 3 (1.6%) and 3 (1.6%) patients, respectively. AMR occurred more frequently in recipients with class II dnDSA than in those without dnDSA (15.0% vs. 1.6%, P = 0.001). However, because of the limited number of overall events, formal multivariable analyses for these clinical outcomes were not performed.

**Table 1:** Baseline characteristics of patients with and without class II dnDSA.

Characteristics	Class II dnDSA (+) (n = 20)	Class II dnDSA (−) (n = 190)	P-value
Recipient’s age (years), median (IQR)	52.5 (41.3–62.8)	52.0 (41.0–60.3)	0.795
Recipient’s sex, male/female	13/7	113/77	0.631
Primary disease, n (%)			0.531
Diabetes mellitus	6 (30.0)	52 (27.3)	
Glomerular disease	3 (15.0)	35 (18.4)	
IgA nephropathy	1 (5.0)	22 (11.6)	
Nephrosclerosis	4 (20.0)	17 (9.0)	
Others	6 (30.0)	64 (33.7)	
Dialysis period (years), median (IQR)	2.1 (1.3–7.1)	2.9 (1.1–8.4)	0.376
History of sensitization, n (%)	7 (35.0)	87 (45.8)	0.356
Donor’s age (years), median (IQR)	61.0 (52.0–67.3)	59.0 (49.8–66.0)	0.477
Donor’s sex, male/female	7/13	89/101	0.312
Type of donor, n (%)			0.199
Deceased	1 (5.0)	31 (16.3)	
Living related	6 (30.0)	72 (37.9)	
Living unrelated	13 (65.0)	87 (45.8)	
History of pregnancy with donor, n (%)	3 (15.0)	30 (15.8)	0.927
ABO-incompatible, n (%)	7 (35.0)	56 (29.5)	0.608
Preformed DSA positive, n (%)	5 (25.0)	31 (16.3)	0.327
Choice of calcineurin inhibitor, TAC/CsA, n	4/16	58/132	0.326
Cold ischemia time (minutes), median (IQR)	118.5 (96.8–138.8)	109.0 (81.0–146.5)	0.317
History of TCMR, n (%)	2 (10.0)	3 (1.6)	0.019
History of AMR, n (%)	3 (15.0)	3 (1.6)	0.001
Observation period (years), median (IQR)	7.1 (4.5–10.3)	6.6 (3.5–10.2)	0.571

AMR, antibody-mediated rejection; Class II dnDSA, *de novo* donor-specific antibody against HLA class II; CsA, cyclosporine; DSA, donor-specific antibody; IgA, immunoglobulin A; IQR, interquartile range; TAC, tacrolimus; TCMR, T-cell–mediated rejection.

Immunological characteristics are summarized in **Table 2**. No significant differences were observed in HLA allele mismatch, EpMM load, PIRCHE-T2 score, or HLA-EMMA load at the HLA-A, -B, or -C loci. At the HLA-DR locus, only EpMM load differed significantly between the groups, whereas HLA allele mismatch, PIRCHE-T2 score, and HLA-EMMA load did not. In contrast, at the HLA-DQ locus and across HLA class II (DR + DQ), EpMM load, PIRCHE-T2 score, and HLA-EMMA load were all significantly higher in patients with class II dnDSAs than in those without. The distributions of class II molecular mismatch loads are shown in **Supplementary Figure 1**. Although median values were higher in the dnDSA-positive group, partial overlap between the two groups was observed. The proportion of *CXCR5* rs3922 G allele carriers was significantly higher in the class II dnDSA-positive group. In contrast, the frequency of the *CTLA4* rs231775 GG genotype did not differ significantly between groups. Nevertheless, the combined genotype (Tfh high) was significantly more prevalent in the class II dnDSA-positive group. Genotype frequencies for CXCR5 rs3922 and CTLA4 rs231775 were consistent with Hardy–Weinberg equilibrium (P = 0.681 and P = 0.662, respectively), indicating no significant deviation from expected genotype distributions.

**Table 2:** Immunological characteristics of patients with and without class II dnDSA.

Variables	Class II dnDSA (+)	Class II dnDSA (−)	P-value
	(n = 20)	(n = 190)	
HLA allele mismatch, n			
A (0: 1: 2)	6: 9: 5	28: 111: 51	0.202
B (0: 1: 2)	1: 10: 9	7: 97: 86	0.958
C (0: 1: 2)	1: 12: 7	22: 102: 66	0.654
DRB1 (0: 1: 2)	0: 9: 11	19: 97: 74	0.194
DQB1 (0: 1: 2)	0: 9: 11	35: 100: 55	0.020
Antibody-verified EpMMs, median (IQR)			
EpMM-A	4.5 (0.0–7.0)	5.0 (1.0–7.0)	0.889
EpMM-B	2.5 (1.0–5.0)	3.0 (2.0–4.3)	0.709
EpMM-C	2.0 (1.0–3.0)	2.0 (0.0–3.0)	0.236
EpMM-DR	5.0 (3.3–13.0)	5.0 (2.0–8.0)	0.013
EpMM-DQ	6.0 (4.0–8.0)	2.0 (0.0–5.0)	<0.001
EpMM class II load	13.0 (8.0–18.8)	8.0 (4.0–12.0)	<0.001
PIRCHE-T2 score, median (IQR)			
PIRCHE-A	17.0 (0.0–35.5)	23.0 (7.0–32.0)	0.610
PIRCHE-B	9.0 (6.0–18.8)	12.0 (6.0–22.0)	0.662
PIRCHE-C	14.0 (7.0–24.0)	15.0 (7.0–24.0)	0.686
PIRCHE-DR	20.5 (13.5–31.5)	19.5 (7.8–35.0)	0.955
PIRCHE-DQ	65.0 (41.8–85.5)	27.0 (9.0–61.0)	<0.001
PIRCHE class II score	79.5 (64.0–111.5)	57.0 (24.3–85.0)	0.005
Solvent-accessible amino acid mismatches by HLA-EMMA, median (IQR)			
EMMA-A	10.5 (0.0–17.3)	10.0 (5.7–17.0)	0.620
EMMA-B	7.5 (3.3–10.0)	7.0 (4.0–10.0)	0.934
EMMA-C	4.5 (2.0–9.0)	4.0 (1.0–7.0)	0.181
EMMA-DR	14.0 (6.5–23.8)	13.0 (3.0–18.0)	0.055
EMMA-DQ	38.5 (18.3–46.5)	11.0 (3.0–32.3)	<0.001
EMMA class II load	46.5 (31.3–70.0)	25.0 (10.8–48.0)	<0.001
Tfh-related SNP status, n (%)			
*CXCR5* rs3922 G allele carrier	12 (60.0)	69 (36.3)	0.039
*CTLA4* rs231775 GG	11 (55.0)	71 (37.4)	0.124
Tfh high	7 (35.0)	25 (13.2)	0.010

Class II dnDSA, *de novo* donor-specific antibody against HLA class II; CTLA-4, cytotoxic T- lymphocyte–associated antigen 4; CXCR5, C-X-C motif chemokine receptor 5; EMMA, HLA Epitope Mismatch Algorithm; EpMM, eplet mismatch; IQR, interquartile range; PIRCHE-T2, Predicted Indirectly ReCognizable HLA Epitopes–T cell model; SNP, single-nucleotide polymorphism; Tfh, T follicular helper; Tfh high, Tfh-prone immunogenetic profile (CXCR5 rs3922 G allele carrier plus CTLA4 rs231775 GG genotype); A, B, C, DR, and DQ represent HLA-A, -B, -C, -DR, and -DQ loci, respectively.

### 3.3 Association between molecular mismatch loads and class II dnDSA development

Receiver operating characteristic curve analyses identified optimal thresholds for EpMM class II (≥7), PIRCHE-T2 class II (≥51), and HLA-EMMA class II (≥28) using the Youden index. These thresholds were subsequently used for exploratory risk stratification analyses in the present cohort, with HLA-EMMA showing the highest discriminative performance (AUC 0.75 vs. 0.70 for both EpMM and PIRCHE-T2; **Supplementary Figure 2**). To complement the threshold-based analyses, continuous-variable analyses were additionally performed per SD increase. All three molecular mismatch metrics remained significantly associated with class II dnDSA development, with HLA-EMMA showing the strongest association (OR, 2.51; 95% CI, 1.58–4.18, P < 0.001; **Supplementary Table 1**).

**Figure 1:**
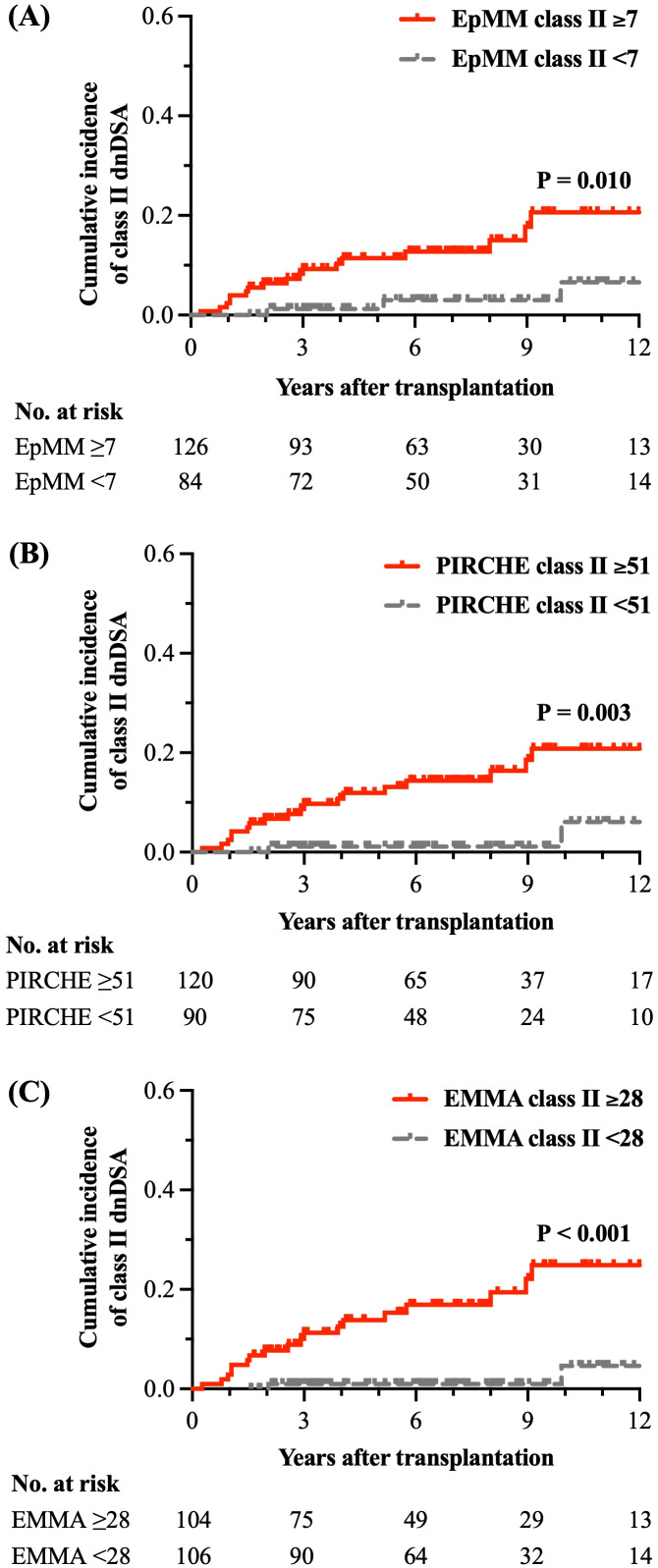
Association between donor–recipient molecular mismatch and risk of class II dnDSA development. Kaplan–Meier curves show the cumulative incidence of class II dnDSA following kidney transplantation stratified by EpMM class II load (≥7 vs. <7) **(A)**, PIRCHE class II score (≥51 vs. <51) **(B)**, and HLA-EMMA class II load (≥28 vs. <28) **(C)**, with log-rank P = 0.010, P = 0.003, and P < 0.001, respectively. Class II dnDSA, *de novo* donor-specific antibody against HLA class II; EpMM, antibody-verified eplet mismatch; PIRCHE, Predicted Indirectly ReCognizable HLA Epitopes; HLA-EMMA, HLA Epitope Mismatch Algorithm.

Kaplan–Meier analysis demonstrated a significantly higher cumulative incidence of class II dnDSA in patients with elevated molecular mismatch loads (EpMM ≥ 7, PIRCHE-T2 ≥ 51, HLA-EMMA ≥ 28) than that in those with lower loads (log-rank P = 0.010, P = 0.003, and P < 0.001, respectively; **Figure 1**). Collinearity assessment demonstrated strong correlations among EpMM, PIRCHE-T2, and HLA-EMMA (Spearman’s ρ = 0.776–0.891; all P < 0.001), indicating substantial overlap among molecular mismatch measures. Because simultaneous inclusion of these variables in a multivariable model could introduce multicollinearity and unstable estimates, the primary multivariable analysis was restricted to a parsimonious model including HLA-EMMA class II and Tfh-high status only. This approach resulted in an EPV ratio of 10 (20 events/2 variables).

**Figure 2:**
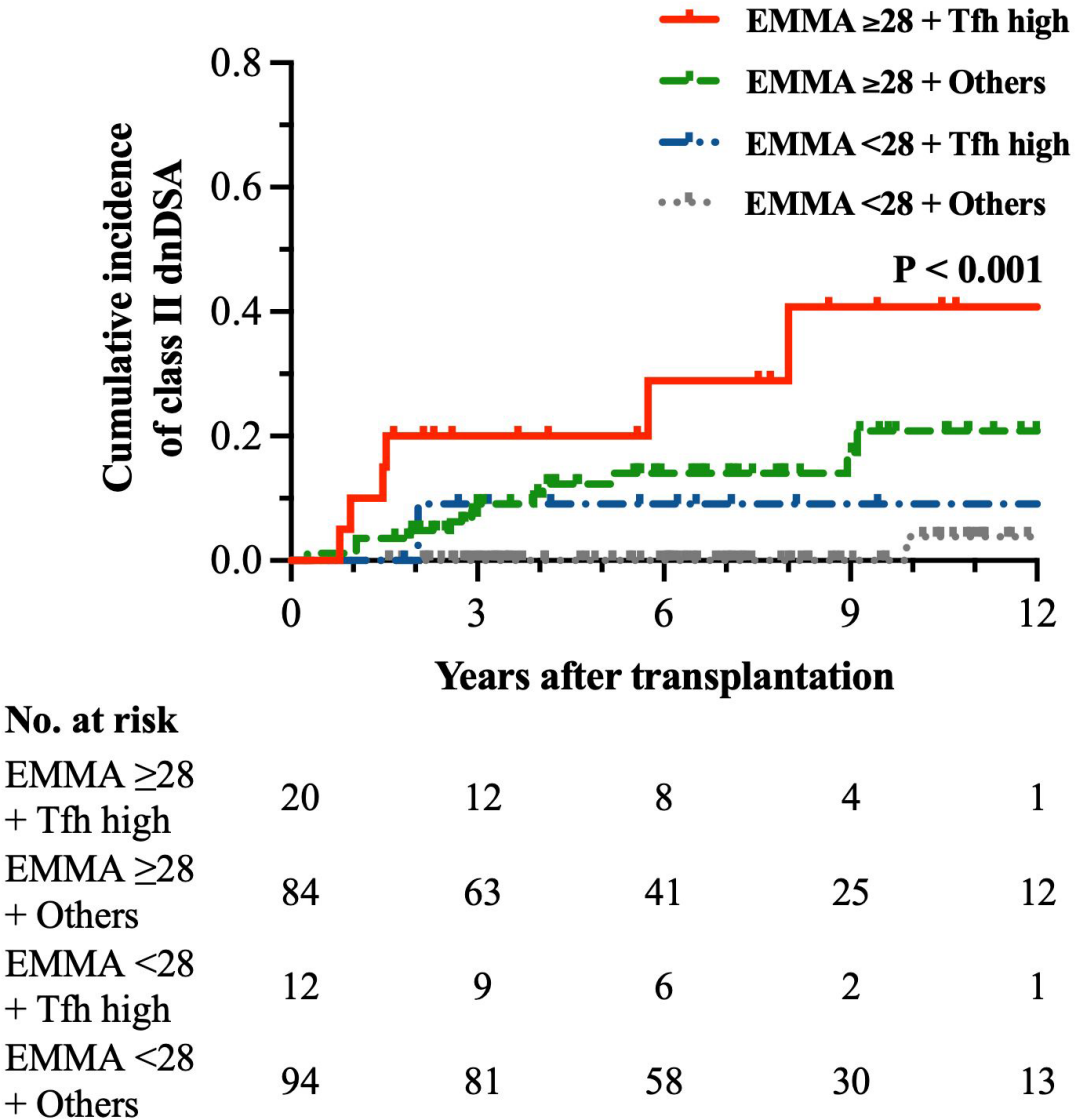
Integrated risk stratification of class II dnDSA development by donor–recipient molecular mismatch and recipient immunogenetic background. Kaplan–Meier curves show the cumulative incidence of class II dnDSA following kidney transplantation stratified according to the combination of HLA-EMMA class II load (≥28 vs. <28) and Tfh-related SNP status (Tfh high: CXCR5 rs3922 G allele carrier plus CTLA4 rs231775 GG genotype vs. others) (log-rank P < 0.001). Class II dnDSA, *de novo* donor-specific antibody against HLA class II; HLA-EMMA, HLA Epitope Mismatch Algorithm; SNP, single-nucleotide polymorphism; Tfh, T follicular helper.

In Firth penalized multivariable logistic regression analysis, HLA-EMMA class II ≥28 (OR, 8.21; 95% CI, 2.49–41.98; P < 0.001) and Tfh-high status (OR, 3.01; 95% CI, 1.04–8.35; P = 0.043) remained significantly associated with class II dnDSA development (**Table 3**). For transparency, results from the exploratory four-variable model, including EpMM, PIRCHE-T2, HLA-EMMA, and Tfh-high status, are presented in **Supplementary Table 2**. Furthermore, combining HLA-EMMA class II load with Tfh-related SNP status enabled further stratification of dnDSA risk (P < 0.001; **Figure 2**).

**Table 3:** Risk factors for *de novo* DSA against HLA class II based on univariable and multivariable analyses.

Variables	Univariable analysis			Multivariable analysis		
	OR	95% CI	P-value	OR	95% CI	P-value
EMMA class II ≥28	8.94	2.73–45.60	<0.001	8.21	2.49–41.98	<0.001
Tfh high	3.61	1.29–9.51	0.016	3.01	1.04–8.35	0.043

ORs, 95% confidence intervals, and P values were estimated using Firth penalized logistic regression because of the limited number of class II dnDSA events.

CI, confidence interval; DSA, donor-specific antibody; EMMA, HLA Epitope Mismatch Algorithm; EpMM, eplet mismatch; OR, odds ratio; PIRCHE, Predicted Indirectly ReCognizable HLA Epitopes; Tfh high, T follicular helper-prone immunogenetic profile (CXCR5 rs3922 G allele carrier plus CTLA4 rs231775 GG genotype).

Sensitivity analyses excluding ABO-incompatible recipients and recipients with preformed DSA demonstrated that the association between HLA-EMMA class II ≥28 and class II dnDSA development remained directionally consistent with the primary analysis. Although the magnitude of association for Tfh-high status was attenuated in these restricted analyses, the overall direction of association remained unchanged (**Supplementary Table 3**). After exclusion of ABO-incompatible recipients, HLA-EMMA class II ≥28 remained independently associated with class II dnDSA development (OR, 10.36; 95% CI, 2.39–96.92). Similar findings were observed after exclusion of recipients with preformed DSA (OR, 10.73; 95% CI, 2.54–99.53).

**Supplementary Table 4** and **S5** summarize the baseline characteristics stratified by HLA-EMMA class II load and Tfh-related SNP status, respectively. The HLA-EMMA ≥ 28 group had a higher proportion of living unrelated donors and a younger donor age, whereas no significant differences were observed across SNP-based groups. No significant association with graft survival was observed for class II dnDSA development, HLA-EMMA class II load, or Tfh-related SNP status (**Supplementary Figure 3**).

## 4 Discussion

In this study, we investigated the interplay between donor-derived HLA molecular disparity and recipient immunogenetic background in relation to humoral alloimmune responses after KT. We demonstrated that higher molecular mismatch loads, particularly HLA-EMMA class II loads, were associated with an increased risk of class II dnDSA development. Furthermore, recipient polymorphisms related to Tfh cell biology were independently associated with dnDSA formation. These findings support a model in which both donor antigenic disparity and recipient T-cell–dependent immune responsiveness may contribute to alloantibody production.

Several molecular mismatch algorithms have been developed to refine the quantification of donor–recipient immunogenic differences beyond conventional HLA matching (33–35). Among these, HLA-EMMA demonstrated the strongest association with dnDSA development in our cohort, suggesting that physicochemical metrics may better approximate the structural features governing B-cell receptor recognition and antibody binding than do conventional epitope-based approaches. These findings support the concept that higher-resolution characterization of molecular antigenicity may improve immunological risk stratification after KT.

However, donor-derived antigenicity alone is unlikely to fully explain the heterogeneity of humoral alloimmune responses after KT. Recipient-dependent immune regulation, particularly Tfh cell function, may represent an additional critical determinant of dnDSA development. Because Tfh cells are central regulators of germinal center–dependent antibody maturation (7, 21, 22), interindividual variability in Tfh cell differentiation, trafficking, and function may substantially influence the magnitude and quality of alloantibody responses. Our findings support this concept by demonstrating that polymorphisms in CXCR5 and CTLA-4 are associated with class II dnDSA development. CXCR5 is essential for the migration of Tfh cells into B-cell follicles, where it facilitates interactions with antigen-specific B cells within germinal centers (36). CTLA-4 acts as a negative regulator of T-cell activation and plays a key role in maintaining immune homeostasis. Genetic variations affecting CTLA-4 expression or function may lower the activation threshold of T cells and enhance Tfh-mediated B-cell help (37, 38). Together, these polymorphisms may define a host immunogenetic context that is associated with robust germinal center responses and alloantibody generation following alloantigen exposure.

Neither donor-derived antigenicity nor recipient immune responsiveness alone fully explains dnDSA development. Although higher molecular mismatch loads were associated with increased dnDSA risk, partial overlap in molecular mismatch distributions was observed between recipients with and without dnDSAs, indicating that donor immunogenicity alone does not fully account for alloantibody development. Similarly, although PIRCHE-T2 scores are designed to reflect indirectly recognizable T-cell epitopes, they did not retain independent significance in the multivariable analysis. This apparent discrepancy suggests that current epitope-based models may incompletely capture the functional variability of recipient T-cell responses. In contrast, recipient immunogenetic variation related to Tfh cell function may influence downstream immune processes beyond antigen presentation, including germinal center dynamics and B-cell selection. These findings raise the possibility that integrating structural measures of antigenicity with host immunogenetic background may provide a more comprehensive representation of alloimmune alloantibody risk.

From an immunological perspective, dnDSA development can be viewed as the outcome of a multistep process involving antigen recognition, T-cell activation, germinal center formation, and antibody maturation (1–4). Our results suggest that HLA molecular mismatch primarily contributes to the initial antigenic stimulus. In contrast, recipient immunogenetic factors may contribute to the efficiency and magnitude of T-cell–dependent B-cell responses. This integrated model provides a conceptual framework in which alloantibody formation may be associated not solely with antigenic disparity but also with the interaction between donor-derived signals and host immune regulatory pathways. However, because functional immune parameters, including Tfh-cell activity, germinal center responses, and B-cell maturation, were not directly assessed, these findings should be interpreted as associative rather than mechanistic.

In our cohort, recipients with class II dnDSA demonstrated a higher frequency of AMR, with a numerically increased frequency of biopsy-proven rejection compared with recipients without class II dnDSA. These findings support the clinical relevance of dnDSA development and are consistent with the established association between dnDSA and alloimmune graft injury after kidney transplantation. However, despite these observed associations, the present study may still have been underpowered to detect significant differences in graft survival and other long-term clinical outcomes because of the relatively limited sample size and event number. In addition, longitudinal analyses of renal function trajectories, proteinuria, and chronic histopathological changes such as transplant glomerulopathy were not systematically available and therefore could not be comprehensively evaluated. Accordingly, further longitudinal studies with larger cohorts and systematic clinical follow-up will be necessary to clarify the relationship between molecular mismatch load, Tfh-related immunogenetic variation, and long-term graft outcomes.

This study has several limitations. First, this was a retrospective, single-center study with a limited number of events, which may have affected the robustness of the statistical analyses and limited the generalizability of the findings. In particular, only a small number of patients developed class I dnDSAs; therefore, the present study focused primarily on class II dnDSAs, which are more frequently observed than class I dnDSAs and clinically relevant after KT. In addition, the limited number of clinical outcome events reduced statistical power for evaluating downstream clinical outcomes, including AMR and graft survival. Therefore, the absence of significant associations with graft survival should be interpreted cautiously.

Second, the present cohort included ABO-incompatible recipients and recipients with preformed DSA, both of whom may have distinct immunologic characteristics and frequently undergo desensitization-related interventions. Such treatments may influence B-cell depletion, Tfh–B-cell interactions, and subsequent donor-specific antibody kinetics. Because desensitization-related interventions and immunologically high-risk conditions frequently overlapped within our cohort, it was difficult to fully disentangle their individual contributions to dnDSA development. Although sensitivity analyses demonstrated generally consistent directional associations for HLA-EMMA class II load, attenuation of the observed associations for Tfh-high status suggests that treatment intensity and baseline immunologic risk may have partially influenced these associations. Therefore, residual confounding related to cohort heterogeneity and desensitization-related factors cannot be completely excluded.

Third, an important limitation of this study is the lack of complete high-resolution HLA typing data. Molecular mismatch estimates were generated using inferred allele-level assignments based on haplotype frequencies for Japanese population (27), which may have introduced a non-differential misclassification of donor–recipient HLA incompatibility. This limitation may be particularly relevant for HLA-EMMA because this algorithm relies on allele-level structural information to quantify the molecular mismatch. Therefore, the reported molecular mismatch associations should be interpreted with caution and require confirmation in cohorts with comprehensive and high-resolution HLA typing data.

Fourth, dnDSA ascertainment was subject to several methodological limitations. The definition of dnDSAs was based on a fixed fluorescence threshold, and detailed functional characterization of antibodies was not systematically performed. In addition, although all recipients underwent a predefined antibody surveillance protocol before transplantation, at 3 months after transplantation, and annually thereafter, additional testing was performed in higher immunologic-risk recipients and in cases of clinically indicated graft dysfunction. Consequently, surveillance frequency was not completely uniform across the cohort. More intensive monitoring in higher-risk recipients may have increased the likelihood of earlier dnDSA detection and could therefore have influenced event ascertainment. Because dnDSA persistence required confirmation at least 6 months after initial detection, differences in surveillance intervals may also have affected the timing and ascertainment of dnDSA events. Therefore, surveillance-related detection bias cannot be completely excluded.

Fifth, the cutoff values used for molecular mismatch metrics require cautious interpretation. The thresholds for EpMM class II, PIRCHE-T2 class II, and HLA-EMMA class II were derived and evaluated within the same cohort and are therefore susceptible to optimism bias and limited generalizability. Consequently, these thresholds should be considered exploratory and hypothesis-generating rather than clinically actionable cutoff values. Their primary purpose in the present study was risk stratification within the study population and comparison of molecular mismatch metrics rather than the establishment of definitive thresholds for clinical decision-making. Moreover, continuous-variable analyses demonstrated generally consistent associations between molecular mismatch load and class II dnDSA development, with HLA-EMMA showing the strongest association among the evaluated metrics. These findings provide complementary support for the observed association beyond the selected categorical thresholds. Nevertheless, neither internal validation approaches such as bootstrapping or cross-validation nor external validation was performed because of the limited number of events. Therefore, additional studies in larger independent multicenter cohorts will be necessary to determine whether similar threshold values can be generalized and whether clinically applicable threshold values can ultimately be established. Furthermore, because the present study was not designed to develop a clinically applicable prediction model, calibration analyses and decision-curve analyses were not performed.

In addition, the classification of Tfh-related immunogenetic risk was based on two candidate SNPs selected *a priori* from our previous studies (25, 29) and may not fully capture the complexity of Tfh cell biology. No direct functional assays of *CXCR5* or *CTLA4* expression or activity were performed in this cohort. Furthermore, although this was a predominantly Japanese single-center cohort, residual population stratification cannot be completely excluded. Therefore, the observed associations should be considered hypothesis-generating and require confirmation through larger studies and mechanistic investigations. Despite these limitations, this study provides novel insights into the immunological factors associated with alloantibody development after KT. By integrating donor-derived structural antigenicity with recipient immunogenetic determinants of Tfh cell function, our findings support a model in which both donor antigenicity and host immune responsiveness may contribute to alloimmune responses. This conceptual framework may inform future studies aimed at refining immunological risk assessment and developing targeted strategies to modulate humoral alloimmunity.

In conclusion, donor–recipient HLA molecular mismatch and recipient Tfh-related immunogenetic variation were associated with the development of class II dnDSA after kidney transplantation. Because molecular mismatch estimates were based on inferred allele-level assignments and the proposed thresholds were derived within a single cohort, independent multicenter validation in larger cohorts is required before clinical implementation.

## Data Availability

The datasets presented in this article are not readily available because they contain sensitive clinical and genetic data from human participants and are subject to ethical and privacy restrictions. Requests to access the datasets should be directed to the corresponding author.
