# Supplementary material for: Donor–recipient HLA molecular mismatch and T follicular helper-related genetic variants are associated with dnDSA development after kidney transplantation
Source: Front Immunol. 2026 Jul 6;17:1875029. doi: 10.3389/fimmu.2026.1875029 (PMC13381205; doi:10.3389/fimmu.2026.1875029)
Supplement: Supplementary file 1 [file DataSheet1.pdf]

Supplementary Material

1 Supplementary Figures and Tables

1.1 Supplementary Figures

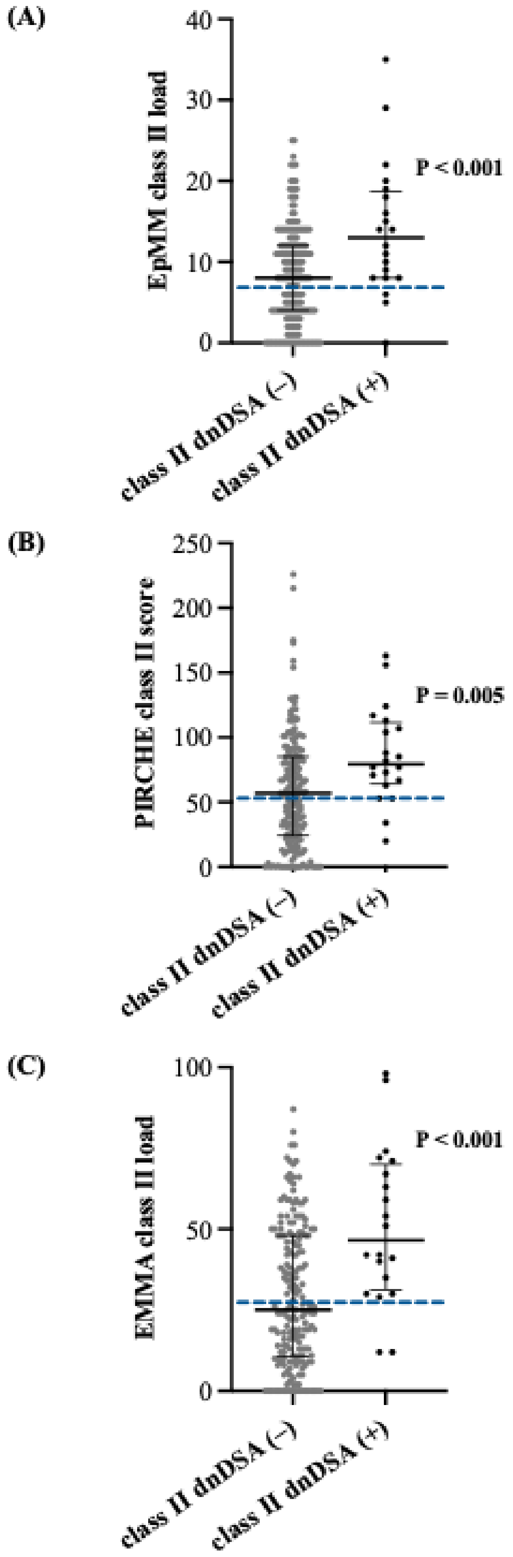

Supplementary Figure S1. Distribution of HLA class II molecular mismatch loads according to class II dnDSA status. Dot plots show the distribution of EpMM class II load (A), PIRCHE class II score (B), and HLA-EMMA class II load (C) in patients with and without class II dnDSA. Horizontal

lines indicate the median, and error bars represent the interquartile range. Dashed lines indicate cutoff values derived from receiver operating characteristic analysis (EpMM ≥7, PIRCHE ≥51, and EMMA ≥28). P-values were calculated using the Mann–Whitney U test.

Class II dnDSA, de novo donor-specific antibody against HLA class II; EpMM, antibody-verified eplet mismatch; PIRCHE, Predicted Indirectly ReCognizable HLA Epitopes; HLA-EMMA, HLA Epitope Mismatch Algorithm.

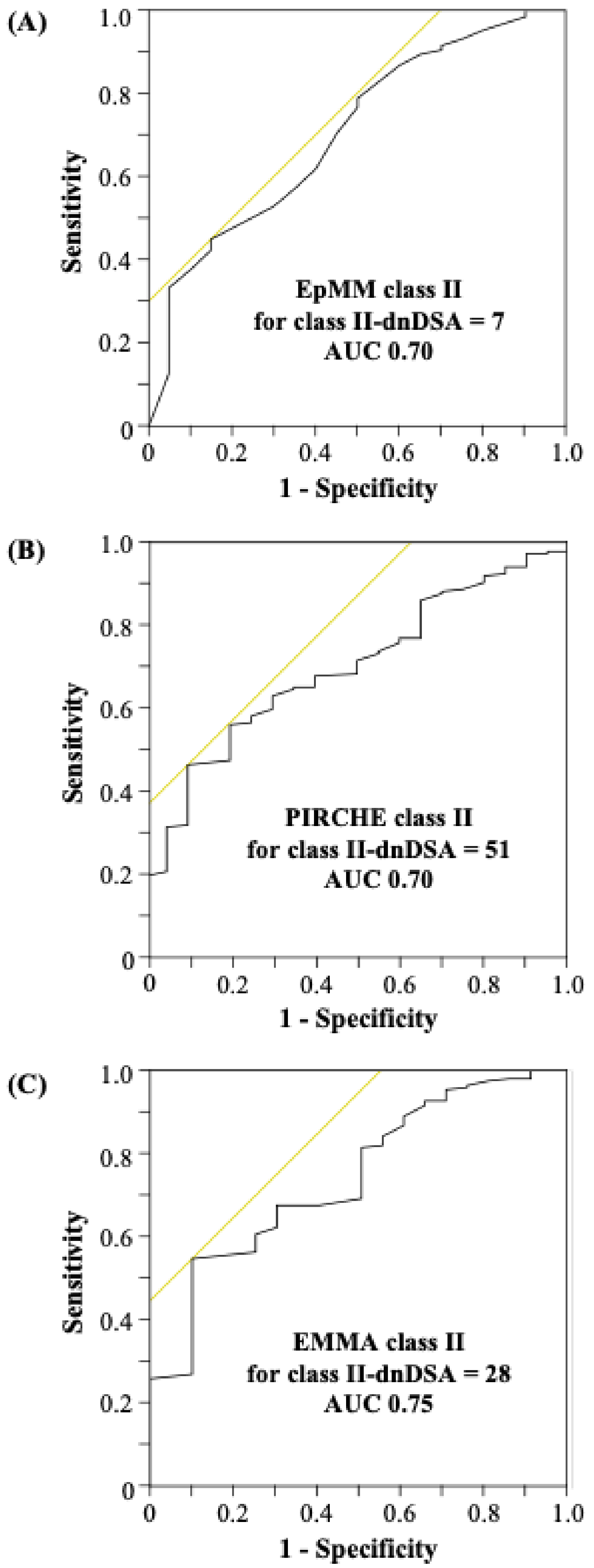
(A)
Sensitivity
1 - Specificity
EpMM class II
for class II-dnDSA = 7
AUC 0.70
(B)
Sensitivity
1 - Specificity
PIRCHE class II
for class II-dnDSA = 51
AUC 0.70
(C)
Sensitivity
1 - Specificity
EMMA class II
for class II-dnDSA = 28
AUC 0.75

Supplementary Figure S2. Receiver operating characteristic (ROC) curves for predicting class II dnDSA development. ROC curves are shown for EpMM class II load (A), PIRCHE class II score (B), and HLA-EMMA class II load (C). The area under the curve (AUC) values were 0.70, 0.70, and 0.75, respectively. The cutoff values derived from the ROC analysis were EpMM ≥7, PIRCHE ≥51, and EMMA ≥28.

Class II dnDSA, de novo donor-specific antibody against HLA class II; EpMM, antibody-verified eplet mismatch; PIRCHE, Predicted Indirectly ReCognizable HLA Epitopes; HLA-EMMA, HLA Epitope Mismatch Algorithm.

(A)

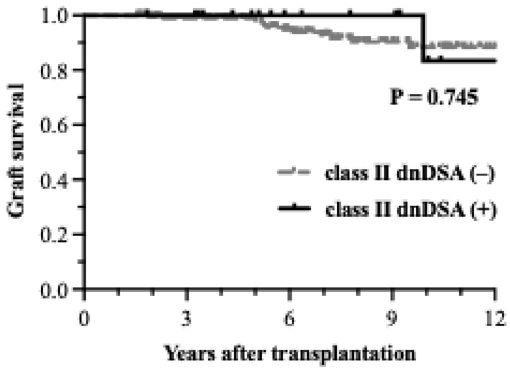

**No. at risk**					
dnDSA (–)	190	155	104	54	23
dnDSA (+)	20	19	11	9	3

(B)

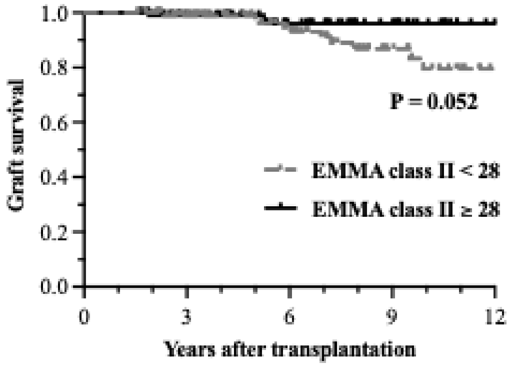

**No. at risk**					
EMMA <28	106	90	61	28	11
EMMA ≥28	104	84	54	35	15

(C)

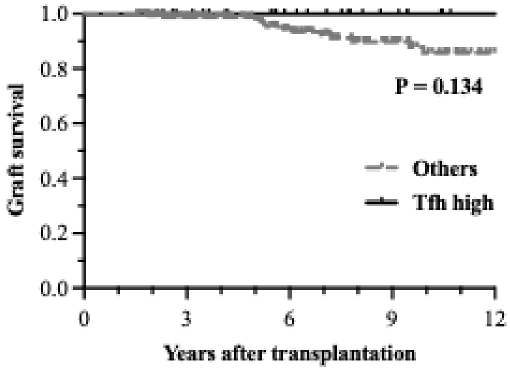

**No. at risk**					
Others	178	148	98	54	23
Tfh high	32	26	17	9	3

Supplementary Figure S3. Kaplan–Meier analysis of graft survival according to immunological risk stratification. Kaplan–Meier curves show graft survival after kidney transplantation stratified by class II dnDSA status (A), HLA-EMMA class II load (≥28 vs. <28) (B), and Tfh-related SNP status (Tfh high vs. others) (C). P-values were calculated using the log-rank test.

Class II dnDSA, de novo donor-specific antibody against HLA class II; HLA-EMMA, HLA Epitope Mismatch Algorithm; SNP, single-nucleotide polymorphism; Tfh, T follicular helper.

1.2 Supplementary Tables

Supplementary Table S1. Continuous-variable univariable Firth penalized logistic regression analysis of class II molecular mismatch metrics (per SD increase) for class II dnDSA development.

Factor	OR	95% CI	P-value
EpMM class II (per SD increase)	2.14	1.40–3.40	<0.001
PIRCHE class II (per SD increase)	1.78	1.17–2.72	0.007
EMMA class II (per SD increase)	2.51	1.58–4.18	<0.001

Univariable analyses were performed using Firth penalized logistic regression. Odds ratios represent the increase in odds of class II dnDSA development per one standard deviation increase in each molecular mismatch metric.

Class II dnDSA, de novo donor-specific antibody against HLA class II; CI, confidence interval; EMMA, HLA Epitope Mismatch Algorithm; EpMM, eplet mismatch; OR, odds ratio; PIRCHE, Predicted Indirectly ReCognizable HLA Epitopes; SD, standard deviation.

Supplementary Table S2. Exploratory four-variable multivariable Firth penalized logistic regression analysis for class II dnDSA development.

Variables	Univariable analysis			Multivariable analysis		
	OR	95% CI	P-value	OR	95% CI	P-value
EpMM class II ≥7	3.72	1.27–14.51	0.015	0.27	0.03–2.47	0.235
PIRCHE class II ≥51	6.39	1.95–32.59	0.001	1.97	0.28–17.93	0.530
EMMA class II ≥28	8.94	2.73–45.60	<0.001	13.22	1.15–268.06	0.036

Tfh high	3.61	1.29–9.51	0.016	3.10	1.06–8.65	0.039

Univariable and multivariable analyses were performed using Firth penalized logistic regression.

CI, confidence interval; DSA, donor-specific antibody; EMMA, HLA Epitope Mismatch Algorithm; EpMM, eplet mismatch; OR, odds ratio; PIRCHE, Predicted Indirectly ReCognizable HLA Epitopes; Tfh high, T follicular helper-prone immunogenetic profile (*CXCR5* rs3922 G allele carrier plus *CTLA4* rs231775 GG genotype).

Supplementary Table S3. Sensitivity analyses after exclusion of ABO-incompatible recipients and recipients with preformed DSA.

Cohort (n/events)	Model	Factor	OR	95% CI	P-value
Full (210/20)	Univ	EMMA ≥28	8.94	2.73–45.60	<0.001
		Tfh high	3.61	1.29–9.51	0.016
	Multi	EMMA ≥28	8.21	2.49–41.98	<0.001
		Tfh high	3.01	1.04–8.35	0.043
ABOi excluded (147/13)	Univ	EMMA ≥28	10.90	2.52–102.05	<0.001
		Tfh high	2.50	0.68–8.10	0.158
	Multi	EMMA ≥28	10.36	2.39–96.92	0.001
		Tfh high	2.18	0.56–7.58	0.246
Preformed DSA excluded (174/15)	Univ	EMMA ≥28	11.38	2.70–105.46	<0.001

Cohort (n/events)	Model	Factor	OR	95% CI	P-value
		Tfh high	3.20	0.97–9.61	0.055
	Multi	EMMA ≥28	10.73	2.54–99.53	<0.001
		Tfh high	2.82	0.82–9.04	0.098
ABOi and Preformed DSA excluded (126/10)	Univ	EMMA ≥28	26.71	3.30–3466.73	<0.001
		Tfh high	1.47	0.26–5.87	0.623
	Multi	EMMA ≥28	26.07	3.24–3377.96	<0.001
		Tfh high	1.32	0.22–5.84	0.733

Univariable and multivariable analyses were performed using Firth penalized logistic regression.

CI, confidence interval; DSA, donor-specific antibody; EMMA, HLA Epitope Mismatch Algorithm; Multi, multivariable analysis; OR, odds ratio; Tfh high, T follicular helper-prone immunogenetic profile (*CXCR5* rs3922 G allele carrier plus *CTLA4* rs231775 GG genotype); Univ, univariable analysis.

Supplementary Table S4. Baseline characteristics stratified by HLA-EMMA class II load.

Characteristics	EMMA class II ≥28 (n = 104)	EMMA class II <28 (n = 106)	P-value
Recipient’s age (years), median (IQR)	53.0 (44.0–60.8)	48.5 (38.0–61.3)	0.084
Recipient’s sex, male/female	61 / 43	65 / 41	0.693

Primary disease, n (%)			0.994
Diabetes mellitus	29 (27.9)	29 (27.4)	
Glomerular disease	18 (17.3)	20 (18.9)	
IgA nephropathy	12 (11.5)	11 (10.4)	
Nephrosclerosis	11 (10.6)	10 (9.4)	
Others	34 (32.7)	36 (33.9)	
Dialysis period (years), median (IQR)	3.2 (1.3–9.6)	2.2 (1.0–7.2)	0.565
History of sensitization, n (%)	49 (47.1)	45 (42.5)	0.497
Donor’s age (years), median (IQR)	56.5 (48.3–63.0)	61.5 (51.0–66.0)	0.010
Donor’s sex, male/female	48 / 56	48 / 58	0.899
Type of donor, n (%)			<0.001
Deceased	16 (15.4)	16 (15.1)	
Living related	25 (24.0)	53 (50.0)	
Living unrelated	63 (60.0)	37 (34.9)	
History of pregnancy with donor, n (%)	21 (20.2)	12 (11.3)	0.077
ABO-incompatible, n (%)	34 (32.7)	29 (27.4)	0.399
Preformed DSA positive, n (%)	17 (16.4)	19 (17.9)	0.762
Choice of calcineurin inhibitor, TAC/CsA, n	30 / 74	32 / 74	0.831
Cold ischemia time (minutes), median (IQR)	109.0 (84.0–145.0)	111.0 (81.0–145.0)	0.630

History of TCMR, n (%)	3 (2.9)	2 (1.9)	0.635
History of AMR, n (%)	3 (2.9)	3 (2.8)	0.981
Class II dnDSA, n (%)	18 (17.3)	2 (1.9)	<0.001
Observation period (years), median (IQR)	6.6 (3.7–10.3)	6.5 (3.5–10.2)	0.939

AMR, antibody-mediated rejection; Class II dnDSA, de novo donor-specific antibody against HLA class II; CsA, cyclosporine; DSA, donor-specific antibody; EMMA, HLA Epitope Mismatch Algorithm; IgA, immunoglobulin A; IQR, interquartile range; TAC, tacrolimus; TCMR, T-cell–mediated rejection.

Supplementary Table S5. Baseline characteristics stratified by Tfh-related SNP status.

Characteristics	Tfh high (n = 32)	Others (n = 178)	P-value
Recipient's age (years), median (IQR)	53.0 (40.5–63.0)	51.5 (41.0–60.3)	0.753
Recipient's sex, male/female, n	16 / 16	110 / 68	0.210
Primary disease, n (%)			0.581
Diabetes mellitus	11 (34.4)	47 (26.4)	
Glomerular disease	4 (12.5)	34 (19.1)	
IgA nephropathy	3 (9.4)	20 (11.2)	
Nephrosclerosis	5 (15.6)	16 (9.0)	
Others	9 (28.1)	61 (34.3)	
Dialysis period (years), median (IQR)	2.9 (0.6–9.9)	2.7 (1.1–7.6)	0.901
History of sensitization, n (%)	14 (43.8)	80 (44.9)	0.901

Donor's age (years), median (IQR)	60.0 (49.3–68.8)	59.0 (50.0–65.0)	0.881
Donor's sex, male/female	17 / 15	79 / 99	0.361
Type of donor, n (%)			0.824
Deceased	6 (18.7)	26 (14.6)	
Living related	11 (34.4)	67 (37.6)	
Living unrelated	15 (46.9)	85 (47.8)	
History of pregnancy with donor, n (%)	4 (12.5)	29 (16.3)	0.587
ABO-incompatible, n (%)	7 (21.9)	56 (31.5)	0.276
Preformed DSA positive, n (%)	5 (15.6)	31 (17.4)	0.805
Choice of calcineurin inhibitor, TAC/CsA, n	10 / 22	52 / 126	0.816
Cold ischemia time (minutes), median (IQR)	114.5 (75.8–153.0)	109.0 (84.0–144.5)	0.776
History of TCMR, n (%)	1 (3.1)	4 (2.3)	0.764
History of AMR, n (%)	3 (9.4)	3 (1.7)	0.016
Class II dnDSA, n (%)	7 (21.9)	13 (7.3)	0.010
Observation period (years), median (IQR)	6.4 (3.5–9.4)	6.6 (3.6–10.5)	0.453

Tfh high was defined as the combined presence of the *CXCR5* rs3922 G allele and *CTLA4* rs231775 GG genotype.

AMR, antibody-mediated rejection; Class II dnDSA, de novo donor-specific antibody against HLA class II; CsA, cyclosporine; CTLA-4, cytotoxic T lymphocyte antigen-4; CXCR5, C-X-C chemokine receptor type 5; DSA, donor-specific antibody; IgA, immunoglobulin A; IQR, interquartile range; SNP, single nucleotide polymorphism; TAC, tacrolimus; TCMR, T-cell–mediated rejection; Tfh, T follicular helper.
